# Themis2/ICB1 Is a Signaling Scaffold That Selectively Regulates Macrophage Toll-Like Receptor Signaling and Cytokine Production

**DOI:** 10.1371/journal.pone.0011465

**Published:** 2010-07-13

**Authors:** Matthew J. Peirce, Matthew Brook, Nicholas Morrice, Robert Snelgrove, Shajna Begum, Alessandra Lanfrancotti, Clare Notley, Tracy Hussell, Andrew P. Cope, Robin Wait

**Affiliations:** 1 Kennedy Institute of Rheumatology Division, Imperial College London, London, United Kingdom; 2 MRC Protein Phosphorylation Unit, University of Dundee, Dundee, United Kingdom; Massachusetts General Hospital and Harvard Medical School, United States of America

## Abstract

**Background:**

Thymocyte expressed molecule involved in selection 1 (Themis1, SwissProt accession number Q8BGW0) is the recently characterised founder member of a novel family of proteins. A second member of this family, Themis2 (Q91YX0), also known as ICB1 (Induced on contact with basement membrane 1), remains unreported at the protein level despite microarray and EST databases reporting Themis2 mRNA expression in B cells and macrophages.

**Methodology/Principal Findings:**

Here we characterise Themis2 protein for the first time and show that it acts as a macrophage signalling scaffold, exerting a receptor-, mediator- and signalling pathway-specific effect on TLR responses in RAW 264.7 macrophages. Themis2 over-expression enhanced the LPS-induced production of TNF but not IL-6 or Cox-2, nor TNF production induced by ligands for TLR2 (PAM3) or TLR3 (poly I∶C). Moreover, LPS-induced activation of the MAP kinases ERK and p38 was enhanced in cells over-expressing Themis2 whereas the activation of JNK, IRF3 or NF-κB p65, was unaffected. Depletion of Themis2 protein by RNA inteference inhibited LPS-induced TNF production in primary human macrophages demonstrating a requirement for Themis2 in this event. Themis2 was inducibly tyrosine phosphorylated upon LPS challenge and interacted with Lyn kinase (P25911), the Rho guanine nucleotide exchange factor, Vav (P27870), and the adaptor protein Grb2 (Q60631). Mutation of either tyrosine 660 or a proline-rich sequence (PPPRPPK) simultaneously interrupted this complex and reduced by approximately 50% the capacity of Themis2 to promote LPS-induced TNF production. Finally, Themis2 protein expression was induced during macrophage development from murine bone marrow precursors and was regulated by inflammatory stimuli both *in vitro* and *in vivo*.

**Conclusions/Significance:**

We hypothesise that Themis2 may constitute a novel, physiological control point in macrophage inflammatory responses.

## Introduction

The engagement of Toll-like receptors (TLRs) on macrophages by pathogen-associated molecular patterns (PAMPs) leads to the generation of pro-inflammatory cytokines and mediators such as TNF, IL-6 and cyclo-oxygenase 2 (Cox2) which not only represent the acute response to infection or trauma but also shape pathogen-specific adaptive immune responses [1]. Many of the signalling components controlling these events are now well characterised and include: Toll-like and IL-1 receptor (TIR) domain-containing adaptors such as MyD88; the kinases IRAK 4 (IL-1 receptor associated kinase 4) and TBK1 (TANK-binding kinase 1); the p38, ERK and JNK MAP kinases; the IkB kinases and members of the AP1, NF-κB and IRF (interferon regulatory factor) families of transcription factors. Despite this, the search for novel TLR signalling components has continued [2] motivated in part by the fact that the above mentioned signalling components are common to multiple TLRs and so only partially explain the receptor specificity apparent in TLR responses [3].

Struck by recent data highlighting the role of tyrosine kinases [4], [5] and tyrosine kinase substrates [6], [7], [8] in TLR signalling we adopted a proteomic approach to recover tyrosine phosphoproteins as putative signalling components from a murine macrophage cell line [9]. This led to the identification of Themis2, a member of a novel protein family named after its founding member, Themis1 (Thymocyte-expressed molecule involved in selection) which is selectively expressed in T lymphocytes [10], [11], [12] and is required for their normal development. In contrast, Themis2 is predominantly expressed in macrophages and B cells (BioGPS, http://biogps.gnf.org). Here, we describe Themis2 protein for the first time, showing that in macrophages Themis2 functions as a signalling scaffold to regulate TLR responses in a receptor- and mediator-selective manner while Themis2 protein levels are responsive to inflammatory stimuli *in vivo* and *in vitro*. We speculate that Themis2 may represent a physiological point of control in macrophage TLR responses.

## Materials and Methods

### Mice

Mouse models of influenza [13] and collagen-induced arthritis [14] were performed as described elsewhere using C57Bl6 mice maintained in specific pathogen free conditions. Husbandry and experimental protocols were carried out in accordance with local institutional (Kennedy Institute of Rheumatology Ethical Procedures Review Committee) and UK Home Office guidelines.

### Cell culture

RAW 264.7 murine macrophages (LGC, Teddington, UK) were cultured in DMEM (Lonza, Wokingham, UK) supplemented with 10% FCS (Biosera, Ringmer, UK) and penicillin/streptomycin (Lonza). Primary human monocyte-derived macrophages (MDMs) were generated by culture (3–5 days) of elutriated human monocytes (80–95% purity) in M-CSF (100 ng/ml; PeproTech, London, UK). Primary murine macrophages were generated from 5 day cultures of bone marrow cells in M-CSF (100 ng/ml).

### Antibodies

Flag reagents were from Sigma (Poole, UK). Anti-phosphotyrosine (4G10) reagents, Vav and Grb2 antibodies were from Upstate Biotechnology (New England Biotech. Hitchin, UK). Antibodies to Lyn, p65 and IRF3 were from Santa Cruz Biotechnology (Insight Biotech. Wembley, UK). The anti-Cox2 antibody came from Transduction Labs (BD Biosciences, Oxford, UK) and antibodies to phosphorylated MAPKs were from Cell Signaling Technology (New England Biotech.). Antibodies to total MAP kinases were gifted by Professor J. Saklatvala (Imperial College London). Two affinity-purified Themis2 rabbit polyclonal antibodies were prepared by standard protocols (Eurogentech, Liege, Belgium) using peptides (CVHKKDRKPNPQTQNS or CEVKVVTKDTRHPTDP) which were respectively specific to murine Themis2, or cross-reactive with human and mouse protein.

### Cloning of Themis2

The Themis2 coding sequence was amplified by PCR from an IMAGE plasmid (clone # 3155889; Geneservice, Cambridge, UK) using primers with a 5′ *Spe1* site (gcgcactagtatggagccggtgccgctgca) and a 3′ *Xba1* site (gcgctctagatcaaatttcttcatagtcatggtcatccatatccggg). The *Spe1/Xba1* digested PCR product was ligated into *Xba1*-linearised pCMV-FLAG4 (Sigma).

### Nucleofection of RAW cells

RAW cells were transfected using the Amaxa system according to the manufacturer's instructions for RAW cells (Lonza).

### Luciferase assays

RAW cells were nucleofected with a firefly luciferase reporter construct containing the 5′ promoter of the human TNF gene [15], relevant expression plasmids and a renilla luciferase construct driven by the Themis2-insensitive thymidine kinase promoter. Firefly and renilla luciferase activities were measured sequentially using a Dual-Glo kit from Promega (Southampton, UK).

### Cytokine ELISAs

Cytokines present in supernatants were captured and detected using appropriate antibody pairs (R and D Systems, Abingdon, UK) and a LabScan fluorescence plate reader.

### Generation of stable transfectants

RAW cells were nucleofected with appropriate expression plasmids (5 µg) and selected (14 days) in complete medium containing G418 (750 µg/ml, Lonza). Resistant cells were expanded and FLAG-tagged protein expression verified by western blot.

### RNA interference

siRNAs (Dharmacon, Epson, UK) targeted to human Themis2 (siRNA A: caauguguacagcaagauu, siRNA B: gaucccgucuacgcuggauu), STAT3 (ccaacaaucccaagaaugu) or a pool of non-targeting siRNAs (cat. # D-001206-13) were mixed (20 min, RT) with Dharmafect 1 (Dharmacon, Thermo Scientific, Epsom, UK) in serum-free OptiMem (Invitrogen). RNA interference (100 nM siRNAs, 2 h) was performed on human MDMs on days 3 and 5 post isolation. On day 7 cells were challenged, or not, with LPS (1 ng/ml, 8 h). Secreted TNF was measured by ELISA and cell lysates analysed by western blot.

### Immunoprecipitations

Detergent extracts of RAW cells (2–5×10^7^ for western blotting or 1×10^9^ for proteomic experiments) were cleared by centrifugation (5 min, 13000×g) pre-incubated (2 h, 4°C) with protein G sepharose (GE healthcare) then with 4G10- or anti-Flag-agarose or anti-Lyn and protein G sepharose beads (2 hr, 4°C, 10 µl beads/plate of cells). Beads were washed (x5) in lysis buffer and bound proteins eluted by boiling in 2X SDS PAGE sample buffer or, for proteomic experiments, with FLAG peptide (1 mM, o/n) and concentrated using a 1D SDS PAGE clean up kit (GE Healthcare, Chalfont St. Giles, UK).

### Statistics

Where appropriate, differences between datasets were analysed using a two-tailed paired Students T test.

### Tandem mass spectrometry

Protein bands were manually excised and digested in-gel with trypsin using an Investigator Progest robot (Genomic Solutions, Huntingdon, UK). Tandem electrospray mass spectra were recorded using a Q-Tof hybrid quadrupole/orthogonal acceleration time of flight spectrometer (Waters, Manchester, UK) interfaced to a Micromass CapLC capillary chromatograph using conditions and parameters described previously [16]. Spectra were searched against the SwissProt database (version 57.11 of 24^th^ November 2009; 512,994 sequences) using a locally installed version of MASCOT (Matrix Science, London UK).

### Tandem mass spectrometric analysis of protein phosphorylation sites

Protein phosphorylation site analysis was performed by LC-MS on an LTQ-orbitrap with multistage activation. Digests were separated on a Biosphere C_18_ trap column (0.1 mm id ×2 mm, Nanoseparations, Holland) connected to a PepMap C18 nano column (75 µm ×15 cm, Dionex Corporation) fitted to a Proxeon Easy-LC nanoflow LC-system (Proxeon, Denmark). The HPLC system was coupled to a linear ion trap-orbitrap hybrid mass spectrometer (LTQ-Orbitrap, Thermo Fisher Scientific Inc) via a nanoelectrospray ion source (Proxeon Biosystems) fitted with a 5 cm Picotip FS360-20-10 emitter. The spray voltage was set to 1.2 kV and the temperature of the heated capillary was set to 200°C. Full scan MS survey spectra (m/z 350–1800) in profile mode were acquired in the Orbitrap with a resolution of 60,000 after accumulation of 500,000 ions. The five most intense peptide ions were fragmented by collision-induced dissociation with multistage activation of the neutral loss of phosphoric acid from the parent ion (neutral loss masses = 49, 32.33 and 24.5 for z = 2,3 and 4). Normalized collision energy of 35%, activation Q 0.250 and activation time 30 ms in the LTQ after the accumulation of 10,000 ions were used with maximal filling times of 1,000 ms for the full scans and 150 ms for the MS/MS scans. Precursor ion charge state screening was enabled and all unassigned charge states, as well as singly charged species, were rejected. The lock mass option was enabled for survey scans to improve mass accuracy. Data were acquired using LTQ-orbitrap 2.5.5 software and analysed using Xcalibur 2.0.7 software. Mascot generic files were created from the raw files using raw2msm (gift from M. Mann, Max Plank Institute for Biochemistry, Martinsried, Germany) and were searched on a local Mascot server (matrixscience.com) against a local database containing the Flag-Themis2 construct sequence with the following criteria. Precursor mass accuracy (10 ppm), MSMS mass accuracy (0.6 Da), enzyme (Trypsin, missed cleavages = 1), Fixed modifications (Carbamidomethylation of cysteine), Variable modifications (Oxidation of methionine, phosphorylation of S, T, Y), minimum ion score = 20.

### Criteria for protein identification

Scaffold (version 2.2.03, Proteome Software Inc., Portland, OR) was used to validate tandem mass spectrometry based peptide and protein identifications. Peptide identifications were accepted if they could be established at greater than 95.0% probability as specified by the Peptide Prophet algorithm [17] and protein identifications were accepted if they contained at least 2 identified peptides and were of greater than 99.0% probability according to the Protein Prophet algorithm [18]. Proteins that contained similar peptides and could not be differentiated based on MS/MS analysis alone were grouped to satisfy the principles of parsimony.

## Results

### Identification of Themis2

In a phosphoproteomic screen of RAW 264.7 cells [9] we identified five peptides (Table S1) from a predicted protein based on a transcript at that point denoted ICB1 (Induced on Contact with Basement membrane) [19], now named Themis2, a relative of the recently identified protein Themis1 [10], [11], [12]. While Themis2 protein has not hitherto been reported Themis2 mRNA is expressed primarily in macrophages and B cells (BioGPS). In contrast Themis1 is predominantly expressed in T lymphocytes. Murine Themis1 and Themis2 share 29% identity and 65% homology at the amino acid level (Fig. S1).

### Themis2 is tyrosine phosphorylated and interacts with Lyn kinase

**Figure 1:**
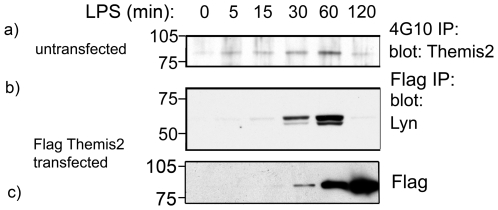
Themis2 is inducibly tyrosine phosphorylated and interacts with Lyn kinase. *Panel a*. RAW cells were challenged with LPS (10 ng/ml) for the indicated period. Phosphotyrosine-containing proteins were recovered with 4G10-agarose, and western blotted with anti-Themis2 antibodies. *Panels b,c*. Anti-Flag immunoprecipitates of LPS-stimulated RAW cells stably expressing Flag-tagged Themis2 were western blotted with anti-Flag-HRP and anti-Lyn kinase. Data shown is representative of three similar experiments.

Themis2 lacks any predicted enzymatic activity or protein interaction domains but, consistent with its identification in anti-phosphotyrosine immunoprecipitates of RAW macrophages [9], it does contain a number of predicted sites of tyrosine phosphorylation (NetPhos 2.0). Most prominent among these is Y660, part of a YEEI motif, a putative peptide substrate for Src or Fes family kinases [20]. Indeed, Y660 was the only detectable site of inducible tyrosine phosphorylation in tandem mass spectrometric analyses of Flag-tagged Themis2 immunoprecipitated from stably transfected RAW cells stimulated with pervanadate (Fig. S2). To examine whether Themis2, like Themis1 in T cells [10], is inducibly tyrosine phosphorylated by physiological stimulation we challenged RAW cells with LPS, immunoprecipitated phosphotyrosine-containing proteins and blotted for endogenous Themis2. As depicted in Figure 1a, LPS induced the time-dependent accumulation of phosphotyrosine on Themis2 without affecting the total levels of Themis2 protein expression (Fig. S3). Once phosphorylated, the YEEI motif containing Y660 is predicted to bind to Src family kinase SH2 (Src homology 2) domains [21]. This prompted us to examine whether Themis2 might interact with a member of the Src kinase family, particularly one, such as Lyn [22], previously linked to TLR4 signalling. Anti-Flag immunoprecipitates, from LPS-stimulated stable RAW cell transfectants expressing Flag-tagged Themis2, were blotted with either anti-Flag-HRP or anti-Lyn kinase (Fig. 1b). LPS stimulation led to the co-immunoprecipitation of Lyn with Themis2 which increased up to 1 hour but returned to baseline by 2 hours, correlating closely with the time course of Themis2 tyrosine phosphorylation (Fig. 1a, Fig. S3). The time-dependent increase in the recovery of Lyn also initially correlated with increased expression of Flag-Themis2 upon LPS challenge (Fig. 1c). As discussed below, this we attributed this up-regulation to the LPS sensitivity of the CMV promoter in the Flag expression vector. However, compared to the 1 h time point, Lyn co-precipitation with Themis2 was lost by 2 h (Fig. 1b) notwithstanding increased levels of Themis2 protein expression (Fig. 1c) suggesting that the interaction of Lyn with Themis2 may be regulated by LPS-stimulation. Confirming the fidelity of the Lyn/Themis2 interaction, Flag-tagged Themis2 was detectable in anti-Lyn immunoprecipitates of LPS-treated, but not resting, Flag-Themis2 expressing RAW cells, nor in Lyn immunoprecipitates of matched numbers of identically treated untransfected RAW cells (Fig. S4).

**Figure 2:**
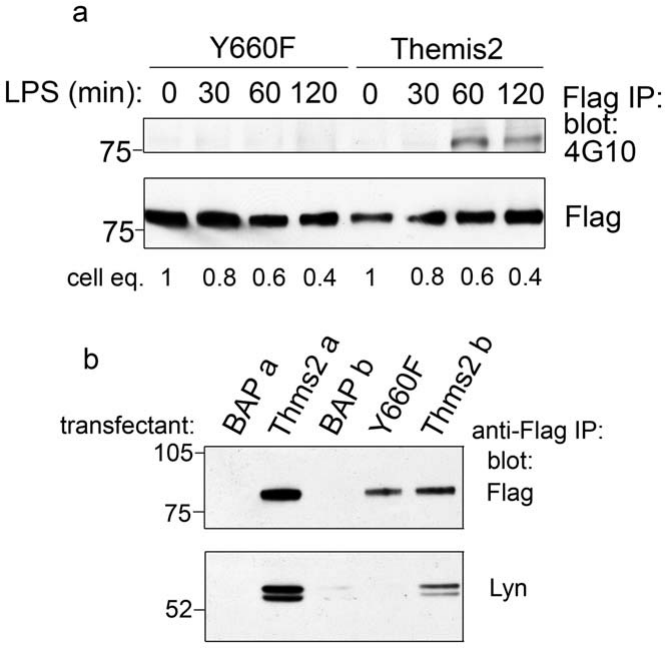
A Y660F mutation of Themis2 inhibits its LPS-induced tyrosine phosphorylation and Lyn interaction. *Panel a.* Flag-tagged Themis2, or a Y660F mutant, was immunoprecipitated from LPS-stimulated (10 ng/ml, 0–120 min) stable RAW cell transfectants. For each time point the relative number of cell equivalents of immunoprecipitated material analysed is indicated. Flag immunoprecipitates were western blotted for phosphotyrosine with 4G10-HRP, stripped and reprobed with anti-Flag-HRP. *Panel b.* Flag-tagged proteins were immunoprecipitated from RAW cells stably expressing Themis2, BAP or the Y660F mutant point mutant of Themis2. For Themis2 and BAP, two independently generated pools of stable transfectants (denoted **a** or **b**) were compared. Immunoprecipitates were western blotted for Flag and Lyn kinase. In each panel a representative of three similar experiments is shown.

To assess whether Y660 might be a site of LPS-inducible tyrosine phosphorylation we generated a point mutant of Themis2 in which Y660 was replaced with phenylalanine (Y660F). Given the LPS-dependent induction of Themis2 expression apparent in Figure 1c noted above, we took care in these experiments to mitigate against this artefact by loading progressively reduced amounts of cell material at later time points. Thus, LPS-induced tyrosine phosphorylation of Flag-tagged wild type Themis2 was readily detectable (Fig. 2a). Consistent with Y660 being phosphorylated in response to LPS, no tyrosine phosphorylation of the Y660F mutant was detected despite similar levels of tagged protein expression.

Speculating that this site might also be involved in the interaction between Themis2 and Lyn, we compared the association of Lyn with wild type Themis2, the Y660F mutant or the control protein bacterial alkaline phosphatase (BAP) (Fig. 2b). In Flag immunoprecipitates of two independently generated pools (labelled a and b) of RAW cells stably over-expressing different amounts of wild-type Themis2, Lyn was successfully co-immunoprecipiated in quantities closely mirroring the levels of Themis2 expression. In cells expressing similar levels of Y660F mutant Themis2, or two independently generated pools of stable BAP transfectants (labelled a and b), no Lyn was detected, consistent with a role for Y660 in the Themis2/Lyn interaction.

### Themis2 interacts with Vav and Grb2

**Figure 3:**
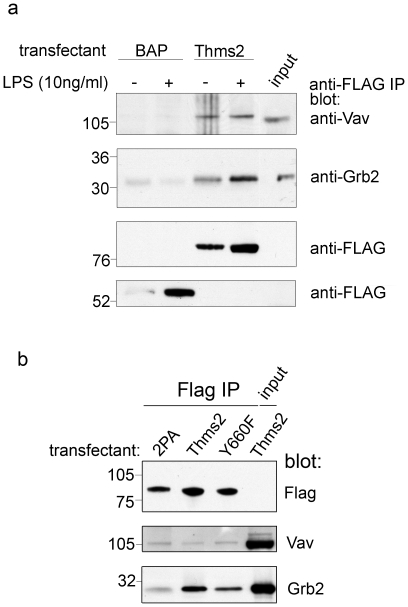
Vav and Grb2 associate with Themis2. *Panel a.* Flag-tagged Themis2 or BAP was immunoprecipitated from matched numbers of stable RAW cell transfectants which had been activated or not with LPS (10 ng/ml, 60 min). Flag-tagged Themis2 and associated Vav or Grb2 were detected by western blot. Vav and Grb2 present in whole cell lysates from Themis2 expressing RAW cells is shown for reference (**input**). *Panel b.* Flag-tagged proteins were immunoprecipitated from matched numbers of stable RAW cell transfectants over-expressing wild type Themis2 (**Thms2**) or its proline (**2PA**) or tyrosine (**Y660F**) mutants. Flag-tagged protein and associated Vav and Grb2 were detected by Western blot. In each case a representative of three similar experiments is shown.

To examine in an unbiased manner whether Themis2 might interact with proteins in addition to Lyn kinase, we immunoprecipitated Flag-tagged Themis2 or BAP, and identified co-immunoprecipitating proteins by SDS-PAGE and tandem mass spectrometry (Fig. S5). Peptides (Table S1) defining the Rho family guanine nucleotide exchange factor (RhoGEF), Vav, were identified in Themis2 but not BAP pull-downs. To test the fidelity of this apparent interaction and to determine whether Grb2, a known interaction partner of Themis1 [12], [23], might also interact with Themis2, similar anti-Flag immunoprecipitations were western blotted for Vav and Grb2. Both Vav and Grb2 immunoreactivity was detectable in pull downs of Themis2 but not BAP (Fig. 3a). In contrast to the interaction between Themis2 and Lyn, its association with Vav was not obviously affected by LPS challenge. Similarly Grb2 was detectable in anti-Flag pull-downs of resting cells but increased with LPS challenge though this was possibly related to increased Themis2 expression in the activated cells. As referred to above, this LPS-dependent up-regulation presumably stems from the CMV promoter in the Flag expression vector, since the control protein, BAP, was similarly up-regulated by LPS-treatment.

Notably, the proline-rich sequence (PPPRPPK) found in Themis1, a potential binding site for SH3 domains such as those found in Grb2 and Vav, is conserved in Themis2. Indeed, an identical sequence is conserved in the Grb2-binding proteins Gab1 and Gab2 and is implicated in their interaction with Grb2 *in vivo* and *in vitro* [24]. To examine whether this sequence might be important in regulating the interactions of Themis2 with Grb2 we generated proline to alanine point mutants (denoted 2PA) of prolines 2 and 4 of the PPPRPPK motif to read P**A**PR**A**PK (mutated sites indicated in bold face). Compared to wild type Themis2, the Y660F and, most strikingly, the 2PA mutant exhibited reduced capacity to interact with Grb2 despite similar levels of tagged protein expression. The interaction of Themis2 with Vav appeared unaffected by either Y660F or 2PA mutations (Fig. 3b).

### Themis2 modulates LPS-induced MAP kinase signalling

**Figure 4:**
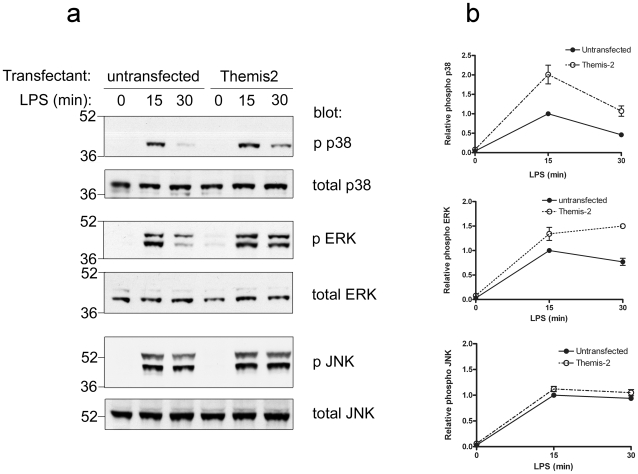
Themis2 over-expression modulates LPS-induced p38 and ERK activation. Parental RAW cells or stable Themis2 transfectants were challenged or not with LPS (10 ng/ml) as indicated. Whole cell extracts were resolved by SDS PAGE and western blotted with antibodies for the phosphorylated MAP kinase indicated then stripped and re-probed with the relevant total MAPK antibody. *Panel a* depicts a representative of four similar experiments which were analysed by scanning densitometry, the means ± sem of which are shown in *panel b*. Values were normalised using total antibody data for each protein and expressed relative to the 15 min time point in parental RAW cells.

**Figure 5:**
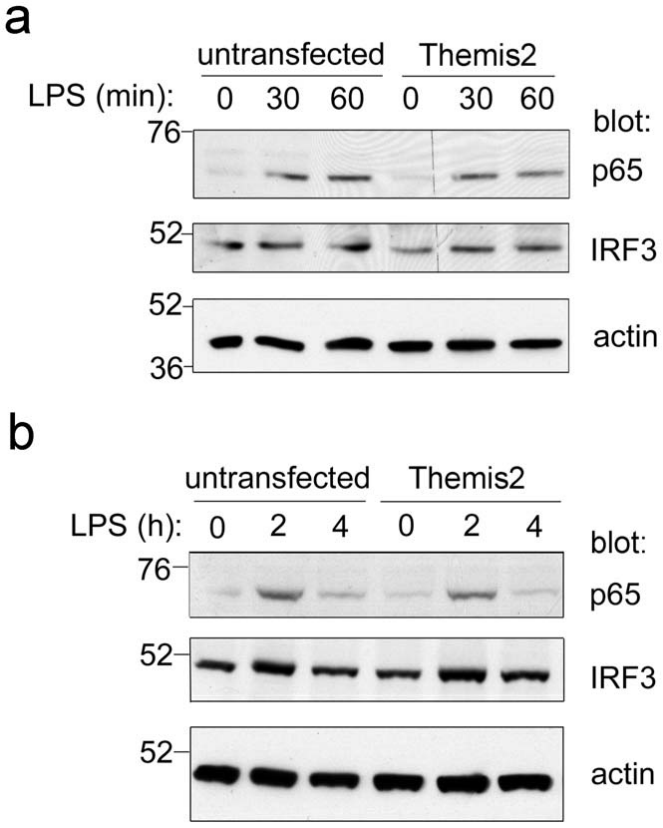
Themis2 over expression has no effect on LPS-induced nuclear localisation of NF-κB p65 or IRF3. Nuclear extracts of parental RAW cells or stable Themis2 transfectants were prepared following LPS challenge for the periods indicated (0–60 min, *panel a*; 0–4 hr, *panel b*), western blotted as shown then stripped and re-probed for actin. A representative of three similar experiments is shown.

Having established interactions between Themis2 and several signalling components we wondered whether Themis2 might impact on signalling pathways in which these proteins are implicated. Vav null macrophages [25] exhibit defects in LPS-induced ERK and p38 MAPK signalling while JNK responses are normal [25]. Grb2 is also known to modulate MAPK signalling [26]. In preliminary experiments we therefore compared the kinetics (0–120 min) of LPS-induced MAP kinase signalling in untransfected RAW cells or stable Themis2 transfectants (Fig. S6). Themis2 over-expression appeared to enhance the longevity and magnitude of LPS-induced p38 activation while marginally inhibiting JNK activation in the same cells. Further investigations focussed on those time points (0–30 minutes) at which differences were most apparent. Notably matching the profile of pathways modulated in Vav-deficient macrophages, Themis2 appeared to enhance the LPS-induced activation of both p38 and ERK compared to parental RAW cells whereas activation of JNK in the same cells was unaffected (Fig. 4a, b). Moreover, the LPS-induced nuclear localisation of NF-κB p65 and IRF3 was unaffected by Themis2 over-expression either acutely (0–60 min, Fig. 5a) or at later time points (2–4 h, Fig. 5b), again mirroring Vav null macrophages [25].

### Themis2 over-expression selectively up-regulates TLR4-mediated TNF production

**Figure 6:**
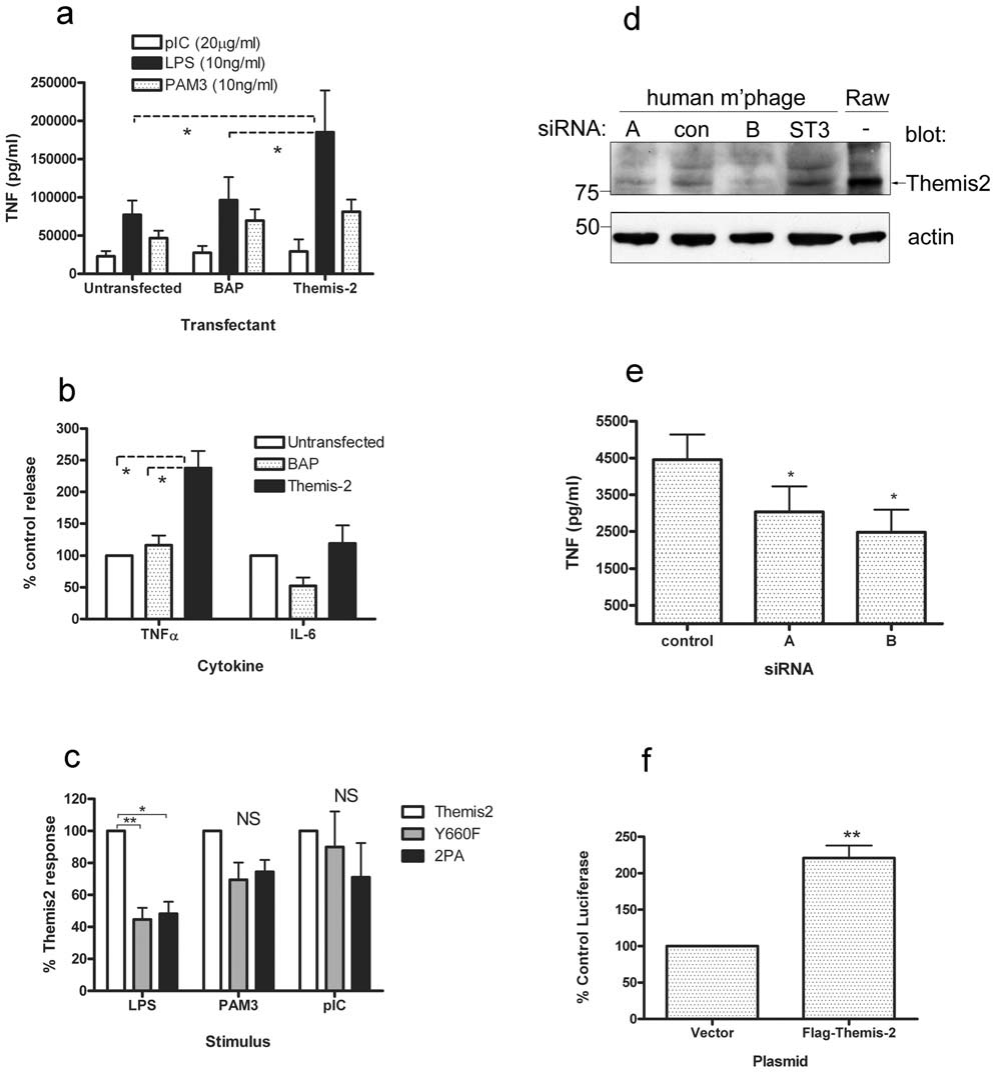
Themis2 regulates TLR-induced cytokine expression. *Panel a*. Parental RAW cells or cells stably over-expressing Themis2 or BAP were challenged (o/n) as indicated. TNF production was measured by ELISA. Data represent the mean ± sem of six experiments. * denotes p<0.05. *Panel b*. Parental or stably transfected RAW cells were challenged (o/n) with LPS and the presence of TNF and IL-6 in supernatants was analysed by ELISA. Data were normalised against values obtained in parental RAW cells and represent the mean ± sem of nine experiments. * denotes p<0.05. *Panel c*. RAW cells stably over-expressing wild-type Themis2 or the 2PA or Y660F mutants were stimulated (o/n) with LPS (10 ng/ml), PAM3 (10 ng/ml) or poly I∶C (20 µg/ml). TNF in cell supernatants was measured by ELISA and expressed relative to the levels generated by wild type Themis2 transfectants. Data represent the mean ± sem of 6 experiments. * denotes p<0.05, ** denotes p<0.005. *Panels d and e.* Primary human macrophages were transfected, or not, on days 3 and 5 with the siRNA indicated (100 nM). On day 7, levels of Themis2 protein and actin were measured by western blot *(panel d)* and LPS-induced generation of TNF was measured by ELISA *(panel e)*. Data represent the mean ± sem of four experiments, * denotes p<0.05. *Panel f*. Parental RAW cells were nucleofected with constructs (2 µg each) encoding renilla luciferase, TNF-firefly luciferase and either Flag-tagged Themis2 or the empty CMV Flag vector. LPS-induced (10 ng/ml, 4 hrs) levels of firefly luciferase activity were normalised against levels of renilla luciferase in the same triplicate wells then expressed as a percentage of the level detected in empty vector-transfected cells. Data represent the mean ± sem of nine experiments. ** denotes p<0.001.

To determine whether these effects on signalling might have functional consequences we compared TNF release in RAW cells stably over-expressing Themis2 or a control protein, BAP, stimulated with ligands for TLR2 (PAM3), TLR3 (poly I∶C) or TLR4 (LPS). Themis2 over-expression enhanced LPS-induced TNF production (approx. 2-fold) without significantly affecting that induced by PAM3 or poly I∶C (Fig. 6a).

To examine whether other inflammatory mediators might be similarly affected LPS-stimulated RAW cells were analysed in parallel for the generation of TNF and IL-6 (Fig. 6b). While Themis2 over-expression enhanced the production of TNF, IL-6 production from the same cells was unaffected. In separate similar experiments, Themis2 over expression also failed to alter the LPS-induced expression of a third inflammatory mediator, Cox2 (Fig. S7).

Having observed differences in the formation of Themis2 signalling complexes resulting from mutations in Y660 or the proline-rich motif (PPPRPPK) we wondered whether these changes might impact on the ability of Themis2 to promote TLR-induced TNF production. Supporting the functional importance of both putative protein interaction sites, the LPS-induced production of TNF protein by RAW cells stably expressing either Y660F or 2PA mutants was significantly reduced (approx. 50% inhibition, p<0.05, n = 6) relative to cells expressing wild type Themis2 (Fig. 6c). Consistent with the previously observed receptor specificity of Themis2 (Fig. 6a), TNF produced in response to PAM3 (TLR2) or poly I∶C (TLR3) was not significantly different in cells expressing wild type Themis2 or its mutants.

To assess whether Themis2 was necessary for LPS-induced TNF production in primary human macrophages we used two siRNAs previously shown to inhibit Themis2 mRNA expression in a human ovarian cancer cell line [27]. As shown in Figure 6d we observed partial reduction (20–50%) of Themis2 protein expression by both siRNAs compared with either a non-targeting control oligo or an oligo targeting the transcription factor STAT3. Consistent with RAW cell over-expression data, LPS-induced TNF release from primary human macrophages partially depleted of Themis2 was significantly inhibited (n = 4, p<0.05) by both siRNAs (Fig. 6e).

While regulated transcription contributes to LPS-dependent control of TNF protein expression in RAW macrophages [28] it is also regulated post transcriptionally [29], largely through AU-rich elements (AREs) present in the 3′ untranslated region (3′UTR) of the mature mRNA transcript. To distinguish between these two modes of regulation we used a luciferase reporter containing the 5′ promoter of human TNF but lacking the 3′ UTR that is required for post transcriptional regulation [15]. Compared with an empty vector control, transient transfection with Flag-tagged Themis2 significantly enhanced (approx. 2-fold) the LPS-induced expression of luciferase (Fig. 6f) suggesting that the effect of Themis2 on TNF protein levels is at least partially attributable to a transcriptional effect.

### Regulation of Themis2 expression

**Figure 7:**
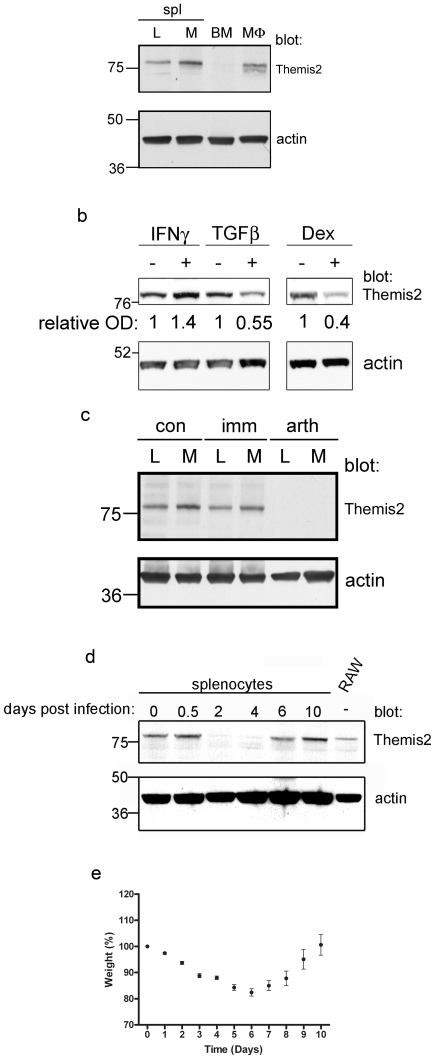
Regulation of Themis2 expression. In panels a–d, detergent extracts of matched numbers of cells were western blotted for Themis2 and actin. Data are representative of three similar experiments. *Panel a*. Whole splenocytes were separated into non-adherent (lymphocyte-enriched, **L**) and adherent (macrophage-enriched, **M**) populations; bone marrow cells undifferentiated (**BM**) or M-CSF-differentiated macrophages (**MΦ**). *Panel b*. RAW cells were treated (48 hrs) with or without IFNγ (10 ng/ml), TGFβ (20 ng/ml) or dexamethasone (0.1 µM). The relative levels of expression in this experiment, corrected for actin expression levels, are presented below each lane. A representative of three similar experiments is presented. *Panel c*. Lymphocyte-enriched (**L**) or macrophage-enriched (**M**) splenocyte populations were recovered as above from control mice (**con**), immunised but disease-free mice (**imm**) or immunised arthritic mice (**arth**) 10 days after immunisation with type II collagen. *Panels d,e*. Mice (5 per time point) were inoculated (50HAU of X31 influenza, nasally) or not, weighed and splenocytes recovered at the indicated time points. Weight (mean±sem) is expressed as a percentage of the value measured at time zero for each animal.

Because Themis2 mRNA is up-regulated during differentiation of HL-60 cells into monocyte-like cells [30] we examined whether Themis2 protein expression might be regulated during differentiation of primary macrophages. Consistent with the mRNA data reported above Themis2 protein was present in both adherent (macrophage-enriched) and non-adherent (lymphocyte-enriched) murine splenocyte populations but was undetectable in undifferentiated bone marrow cells. *In vitro* differentiation of these precursors into macrophages, by culture in the presence of M-CSF, induced expression of Themis2 (Fig. 7a).

Themis2 mRNA expression is also reported to be regulated by IFNγ [27] or estrogen [31] treatment of ovarian cancer cell lines. Similarly, Themis2 protein was up-regulated by treatment (48hrs) of RAW macrophages with pro-inflammatory stimuli (IFNγ, mean fold increase±sem: 1.58±0.09, n = 3) but inhibited by anti-inflammatory stimuli (TGFβ, 0.57±0.04, n = 3; dexamethasone, 0.47±0.16, n = 3) without any detectable effect on actin expression in the same cells (Fig. 7b).

The regulation of Themis2 protein levels by pro- and anti-inflammatory stimuli *in vitro* prompted us to examine whether similar regulation of Themis2 expression might occur during *in vivo* inflammatory responses. We measured Themis2 protein expression in adherent (macrophage-enriched) or non-adherent (B cell-enriched) splenocyte populations in a murine model of arthritis. Using a collagen-induced arthritis model [14], mice immunised with collagen but failing to develop arthritis exhibited no change in Themis2 expression. In contrast, in those mice developing arthritic symptoms following collagen immunisation Themis2 expression in either adherent or non-adherent splenocytes was abolished (Fig. 7c). Similarly, in an infectious disease model, infection of mice with influenza virus led to a complete but transient loss of Themis2 expression in whole splenocytes (Fig. 7d) that broadly coincided with peak levels of weight loss (Fig. 7e), an indirect measure of lung inflammation [13].

## Discussion

Themis1 is the recently identified founder member of a family of five proteins defined on the basis of two repeats of a CABIT domain [11] and is selectively expressed in T cells [10], [11], [12]. Themis2 is a previously uncharacterised member of this family whose mRNA is selectively expressed in B cells and macrophages. The data presented here describe Themis2 protein for the first time and characterise its function in macrophages.

We demonstrate that Themis2 behaves as a regulator of TLR signalling with an unusually specific mode of action, controlling the generation of TNF induced by TLR4 engagement without affecting TNF generated in response to ligands for TLRs 2 or 3, nor the TLR4-dependent production of other inflammatory mediators such as IL-6 or Cox2. Such specificity is in marked contrast to the majority of known TLR signalling components which are common to multiple TLRs [1], [2]. The molecular basis of this specificity remains to be clarified but may be explained by its interaction with the RhoGEF Vav. The tyrosine phosphorylation of Vav, linked to activation of its RhoGEF activity [32], is induced following TLR4 engagement on RAW cells [33]. Moreover, contrary to the sharing of signalling components by different TLRs noted above, several RhoGEFs and members of the Rho/Rac family GTPases they regulate, have been shown to modulate TLR signalling in a receptor or mediator-selective manner. These include RhoA [34] and the RhoGEFs Def6 [35], AKAP13 [36] and Vav itself [25]. Further supporting a role for Vav in the effects of Themis2, the deficits resulting from genetic deletion of all three Vav proteins strikingly mirror the events modulated by over-expression of Themis2. Thus, LPS-induced TNF responses are deficient in Vav null macrophages despite normal IL-6 and Cox2 production [25]. This parallel extends to the signalling pathways affected: LPS-induced p38 and ERK MAP kinase activation are inhibited in Vav null macrophages while JNK and NF-κB responses are spared [25]. Similarly, RAW cells over-expressing Themis2 exhibit enhanced LPS-induced p38 and ERK activation despite normal JNK responses and nuclear localisation of NF-κB and IRF3.

How might the noted effects on p38 and ERK signalling modulate TNF production? While the role of p38 and ERK in LPS-induced TNF expression is now well established they are believed regulate both overlapping and distinct elements of the process. ERK activation is associated with the regulation of TNF transcription, possibly via the phosphorylation of AP1 family members such as Egr1 [37], [38]. Thus, it is possible that the up-regulation of transcription of the TNF-luciferase reporter by Themis2 (Fig. 6f) is causally linked to the up-regulation of LPS-induced ERK phosphorylation (Fig. 4a, b). p38 activation modulates TNF protein expression by regulating the expression and function of the ARE-binding protein Tristetraprolin (TTP) and thereby robustly enhancing the stability of TNF message [29], [39], [40]. However, ERK also regulates TTP protein stability [39] and ERK and p38 activities can jointly regulate TNF mRNA stability [41]. Therefore it is entirely possible that such post-transcriptional mechanisms also contribute to the observed effects of Themis2 on TNF protein expression. It will therefore be of interest, in future experiments, to examine whether Themis2 has any effect on TNF mRNA stability.

The correlation in the pathways and cytokines modulated by Vav deletion and Themis2 over-expression suggest that the functional impact of Themis2 on these events is mediated primarily through its interaction with Vav. The molecular basis of this interaction is now the focus of ongoing work in our laboratory. Vav contains an SH2 domain and two SH3 domains which could respectively mediate interaction through tyrosine phosphorylation of Themis2, for example at Y660, or through proline-rich sequences such as that common to both Themis1 and 2 (PPPRPPK). Our data suggest that the interaction with Vav is both constitutive (Fig. 3a) and independent of Y660 (Fig. 3b), arguing against an association mediated by the Vav SH2 domain and inducible tyrosine phosphorylation on Themis2. In addition, alanine point mutants of prolines 2 and 4 (indicated in bold face) of the P**P**PR**P**PK motif had no obvious effect on the Vav/Themis2 interaction despite inhibiting its interaction with Grb2 (Fig. 3b, discussed below) arguing against this motif as a Vav binding site. However, Themis2 contains a number of motifs conforming to the patterns (RxPxxP, PxxPxR or PxxP) reported for SH3 ligands, any one of which could mediate the interaction with Vav. Mutational analyses of these putative SH3-binding motifs, now underway in our laboratory, will help to define the site of interaction between Themis2 and Vav and address its functional significance.

While further study is required to clarify the role of Vav, our current data do support a role for other protein/protein interactions in Themis2 function. The Src family kinase Lyn appears to interact with Themis2 in a manner that correlates with the tyrosine phosphorylation of Themis2 (Fig. 1a) and is at least partially dependent on Y660 since the interaction is lost in the Y660F Themis2 mutant (Fig. 2). Using tandem mass spectrometry we directly demonstrated the tyrosine phosphorylation of Y660 in vivo, albeit using a robust chemical stimulus, pervanadate. The same phosphopeptide was not detectable in a similar study using Themis2 derived from LPS-treated RAW cells (data not shown), probably a reflection the lower stoichiometry achieved using a physiological stimulus. However, the fact that mutation of Y660 abolishes the LPS-induced tyrosine phosphorylation of Themis2 (Fig. 2) strongly suggests that this site is indeed targeted during LPS signalling. Together, these data suggest a model in which LPS drives the tyrosine phosphorylation of Themis2 at Y660 which allows Lyn to bind via its SH2 domain. The functional importance of this interaction is supported by the observation that the ability of Themis2 to promote LPS-induced TNF production is significantly inhibited by mutation of Y660 (Fig 6c). How might the loss of Lyn binding impact upon LPS signalling? As discussed above Vav is known to be a target of LPS-regulated tyrosine phosphorylation [33], and this controls its RhoGEF activity [32]. Indeed, the Src family kinase Hck, a close relative of Lyn, is reported to phosphorylate Vav following LPS challenge of RAW macrophages [33]. One possibility under active investigation is that by providing a platform on which both proteins are recruited, Themis2 might serve to facilitate the tyrosine phosphorylation of Vav by Lyn.

The data presented here also suggest that, as reported for its relative Themis1 [12], [23], Themis2 interacts with the adaptor protein Grb2. Our data are consistent with a role for the PPPRPPK motif in this interaction since mutation of prolines 2 and 4 in this sequence simultaneously inhibited interaction with Grb2 (Fig. 3b) and the ability of Themis2 to promote LPS-induced TNF release (Fig. 6c). Together these data strongly suggest that the Themis2 protein complex defined here must be retained intact for optimal LPS-induced TNF release. The reported interaction between Grb2 and Themis1 [12] is proposed to involve both SH2 and SH3 domains of Grb2. In this light it is notable that the Y660F mutation of Themis2 also appears to reduce its interaction with Grb2, although not to the extent of the 2PA proline mutant (Fig. 3b). Thus, like the Themis1, the interaction between Themis2 and Grb2 may involve contributions from both proline-rich and phosphotyrosine ligands.

While little literature precedent exists describing a role for Grb2 in TLR signalling, the strategy of clustering together the same group of signalling proteins appears to have been adopted by at least one pathogen to subvert TLR signalling in B cells. The murine gammaherpesvirus 68 (MHV68) protein, M2, interacts with Grb2 [42], Vav, and the Src kinase Fyn [43]. Akin to the effects of Themis2 over-expression reported here, M2 augments the LPS-driven proliferation of B cells and selectively regulates the generation of some inflammatory mediators (e.g. IL-10) but not others (e.g. RANTES) [44]. While MHV68 M2 and Themis2 share little amino acid sequence homology they both appear to function as scaffolds for assembling signalling complexes of Vav, Grb2 and members of the Src kinase family and seemingly share the capacity to modulate LPS responses and selectively modulate inducible cytokine production.

Just as Themis1 is required for the development of T cells so Themis2 is induced upon differentiation of macrophages from bone marrow precursors under the influence of M-CSF. It is also of interest that other modulators of macrophage phenotype regulate the level of Themis2 protein expression. We show that IFNγ, a cytokine known to promote inflammatory macrophage responses including TNF production [45], increases the expression of Themis2 protein whereas TGFβ and dexamethasone, which inhibit LPS-induced TNF production [45], reduce Themis2 levels. Since we also demonstrate that *in vivo* immune responses to both inflammatory (CIA) and infectious (influenza) disease are also associated with profound regulation of Themis2 protein levels it is tempting to speculate that Themis2 expression may represent an important physiological point of control by which macrophage inflammatory phenotype and function, and specifically the magnitude of TNF responses, may be dynamically regulated. The mechanisms by which Themis2 protein levels are regulated by inflammatory signals have yet to be investigated but it is worth noting that the 3′UTR of Themis2 contains several AREs associated with the binding of proteins controlling mRNA stability [46]. Indeed one sequence, UUAUUUAUU, found in the Themis2 3′UTR represents the cognate binding site for TTP [47], a post-transcriptional regulator induced by inflammatory stimuli and implicated in the control of the expression of multiple inflammatory genes [48]. It will also be informative to dissect the 5′ promoter region of Themis2 to define potential sites of transcriptional regulation.

In summary, we identify Themis2 as a novel macrophage signalling scaffold that selectively regulates TNF expression downstream of TLR4 and whose expression is itself regulated during macrophage differentiation and *in vivo* models of infectious and inflammatory disease. As such, Themis2 could represent a novel site at which therapies designed to intervene in chronic inflammatory diseases involving TNF, such as rheumatoid arthritis, might usefully be targeted.

## Supporting Information

Table S1 Peptides defining Themis2 and Vav. ^a^ Mascot ion scores reflect the probability (p) of chance identification of the same peptide: scores>29 for the Themis2 search, or >30 for the Vav search, denote p<0.05. ^b^ Scaffold probability reflects the percentage confidence that each peptide sequence identified is a non-random event. The Scaffold calculated probability scores for each protein as a whole was 100%.
(0.03 MB DOC)

Figure S1 Comparison of murine Themis1 and Themis2. Sequences were compared using the alignment tool in the ExPASy proteomics server (http://us.expasy.org).
(0.03 MB DOC)

Figure S2 MS/MS detection of tyrosine phosphorylated Y660. Flag-Themis2 from resting cells or pervanadate-treated cells was isolated by immunoprecipiation, separated by SDS-PAGE and digested with trypsin. The tryptic digests were analysed by LC-MS on an LTQ-orbitrap and the c-terminal peptide HSTmESHLLPDPDmDDHDpYEEI was identified from the pervanadate treated sample (A) with a Mascot ion score of 31 (m represents oxidised methionine and pY represents phosphotyrosine). The extracted ion chromatogram of this phosphopeptide ion (m/z = 913.349) from Themis2 isolated from resting and pervanadate-treated cells is shown (B). Similar changes were observed for the same peptide without oxidation (m/z = 902.686) and with one oxidised methionine (m/z = 908.017) ( data not shown).
(0.33 MB TIF)

Figure S3 LPS-induced tyrosine phosphorylation of endogenous Themis2. Phosphotyrosine-containing proteins were immunoprecipitated from detergent extracts of LPS-treated RAW cells (10 ng/ml, 0–120 min). Immunoprecipitates or input material from each time point was western blotted for Themis2.
(0.16 MB TIF)

Figure S4 Themis2 interacts with Lyn kinase. Untransfected parental RAW cells or cells stably expressing Flag-tagged Themis2 were stimulated (1 hr), or not, with LPS (10 ng/ml). Immunoprecipitations with anti-Flag or anti-Lyn were performed on separate aliquots of the same cleared extracts. Immunoprecipitates were western blotted as indicated. Data depict a representative of three similar experiments.
(0.72 MB TIF)

Figure S5 Identification of Themis2 interacting proteins. Flag-tagged and associated proteins were recovered using Flag-agarose beads, eluted, concentrated, resolved and visualised as described in Methods. Protein bands were digested with trypsin and peptides identified by tandem mass spectrometry (see Supplementary Table 1) and validated using Scaffold software.
(0.27 MB TIF)

Figure S6 Over-expression of Themis2 promotes LPS-induced p38 but not JNK activation. Parental RAW cells or cells stably over-expressing Themis2 were stimulated with LPS (10 ng/ml) for the period indicated. Anti-phosphotyrosine-containing proteins were immunoprecipitated from detergent lysates with 4G10-agarose beads. The presence of total p38 and JNK MAPKs in immunoprecipitates or input material was detected by western blotting. A representative of five similar experiments is shown.
(0.57 MB TIF)

Figure S7 Themis2 over-expression has no effect on LPS-induced Cox2 expression. Matched numbers of parental RAW cells or cells stably over-expressing Themis2 were challenged with LPS (10 ng/ml) for the period indicated and detergent extracts western blotted for Cox2 and actin. Data shown are representative of four similar experiments.
(0.61 MB TIF)

## References

[1] Akira S, Uematsu S, Takeuchi O (2006). Pathogen recognition and innate immunity.. Cell.

[2] Hacker H, Redecke V, Blagoev B, Kratchmarova I, Hsu LC (2006). Specificity in Toll-like receptor signalling through distinct effector functions of TRAF3 and TRAF6.. Nature.

[3] Amit I, Garber M, Chevrier N, Leite AP, Donner Y (2009). Unbiased reconstruction of a mammalian transcriptional network mediating pathogen responses.. Science.

[4] Horwood NJ, Mahon T, McDaid JP, Campbell J, Mano H (2003). Bruton's tyrosine kinase is required for lipopolysaccharide-induced tumor necrosis factor alpha production.. J Exp Med.

[5] Smolinska MJ, Horwood NJ, Page TH, Smallie T, Foxwell BM (2008). Chemical inhibition of Src family kinases affects major LPS-activated pathways in primary human macrophages.. Mol Immunol.

[6] Gray P, Dunne A, Brikos C, Jefferies CA, Doyle SL (2006). MyD88 adapter-like (Mal) is phosphorylated by Bruton's tyrosine kinase during TLR2 and TLR4 signal transduction.. J Biol Chem.

[7] Jefferies CA, Doyle S, Brunner C, Dunne A, Brint E (2003). Bruton's tyrosine kinase is a Toll/interleukin-1 receptor domain-binding protein that participates in nuclear factor kappaB activation by Toll-like receptor 4.. J Biol Chem.

[8] Doyle SL, Jefferies CA, Feighery C, O'Neill LA (2007). Signaling by Toll-like receptors 8 and 9 requires Bruton's tyrosine kinase.. J Biol Chem.

[9] Peirce MJ, Begum S, Saklatvala J, Cope AP, Wait R (2005). Two-stage affinity purification for inducibly phosphorylated membrane proteins.. Proteomics.

[10] Fu G, Vallee S, Rybakin V, McGuire MV, Ampudia J (2009). Themis controls thymocyte selection through regulation of T cell antigen receptor-mediated signaling.. Nat Immunol.

[11] Johnson AL, Aravind L, Shulzhenko N, Morgun A, Choi SY (2009). Themis is a member of a new metazoan gene family and is required for the completion of thymocyte positive selection.. Nat Immunol.

[12] Lesourne R, Uehara S, Lee J, Song KD, Li L (2009). Themis, a T cell-specific protein important for late thymocyte development.. Nat Immunol.

[13] Snelgrove RJ, Goulding J, Didierlaurent AM, Lyonga D, Vekaria S (2008). A critical function for CD200 in lung immune homeostasis and the severity of influenza infection.. Nat Immunol.

[14] Inglis JJ, Simelyte E, McCann FE, Criado G, Williams RO (2008). Protocol for the induction of arthritis in C57BL/6 mice.. Nat Protoc.

[15] Denys A, Udalova IA, Smith C, Williams LM, Ciesielski CJ (2002). Evidence for a dual mechanism for IL-10 suppression of TNF-alpha production that does not involve inhibition of p38 mitogen-activated protein kinase or NF-kappa B in primary human macrophages.. J Immunol.

[16] Peirce MJ, Wait R, Begum S, Saklatvala J, Cope AP (2004). Expression profiling of lymphocyte plasma membrane proteins.. Mol Cell Proteomics.

[17] Keller A, Nesvizhskii AI, Kolker E, Aebersold R (2002). Empirical statistical model to estimate the accuracy of peptide identifications made by MS/MS and database search.. Anal Chem.

[18] Nesvizhskii AI, Keller A, Kolker E, Aebersold R (2003). A statistical model for identifying proteins by tandem mass spectrometry.. Anal Chem.

[19] Treeck O, Strunck E, Vollmer G (1998). A novel basement membrane-induced gene identified in the human endometrial adenocarcinoma cell line HEC1B.. FEBS Lett.

[20] Songyang Z, Carraway KL, Eck MJ, Harrison SC, Feldman RA (1995). Catalytic specificity of protein-tyrosine kinases is critical for selective signalling.. Nature.

[21] Songyang Z, Shoelson SE, Chaudhuri M, Gish G, Pawson T (1993). SH2 domains recognize specific phosphopeptide sequences.. Cell.

[22] Stefanova I, Corcoran ML, Horak EM, Wahl LM, Bolen JB (1993). Lipopolysaccharide induces activation of CD14-associated protein tyrosine kinase p53/56lyn.. J Biol Chem.

[23] Patrick MS, Oda H, Hayakawa K, Sato Y, Eshima K (2009). Gasp, a Grb2-associating protein, is critical for positive selection of thymocytes.. Proc Natl Acad Sci U S A.

[24] Lock LS, Royal I, Naujokas MA, Park M (2000). Identification of an atypical Grb2 carboxyl-terminal SH3 domain binding site in Gab docking proteins reveals Grb2-dependent and -independent recruitment of Gab1 to receptor tyrosine kinases.. J Biol Chem.

[25] Miletic AV, Graham DB, Montgrain V, Fujikawa K, Kloeppel T (2007). Vav proteins control MyD88-dependent oxidative burst.. Blood.

[26] Gong Q, Cheng AM, Akk AM, Alberola-Ila J, Gong G (2001). Disruption of T cell signaling networks and development by Grb2 haploid insufficiency.. Nat Immunol.

[27] Treeck O, Kindzorra I, Pauser K, Treeck L, Ortmann O (2005). Expression of icb-1 gene is interferon-gamma inducible in breast and ovarian cancer cell lines and affects the IFN gamma-response of SK-OV-3 ovarian cancer cells.. Cytokine.

[28] Raabe T, Bukrinsky M, Currie RA (1998). Relative contribution of transcription and translation to the induction of tumor necrosis factor-alpha by lipopolysaccharide.. J Biol Chem.

[29] Brook M, Sully G, Clark AR, Saklatvala J (2000). Regulation of tumour necrosis factor alpha mRNA stability by the mitogen-activated protein kinase p38 signalling cascade.. FEBS Lett.

[30] Treeck O, Odani T, Itoh N, Imai H, Fujita S (2002). Detection of increased icb-1 transcript levels in maturing HL-60 cells: a novel marker for granulocytic and monocytic in vitro differentiation.. Leuk Res.

[31] Bollmann J, Ortmann O, Treeck O (2008). Expression of differentiation-associated gene icb-1 is estrogen-responsive in ovarian and breast cancer cell lines.. J Steroid Biochem Mol Biol.

[32] Crespo P, Schuebel KE, Ostrom AA, Gutkind JS, Bustelo XR (1997). Phosphotyrosine-dependent activation of Rac-1 GDP/GTP exchange by the vav proto-oncogene product.. Nature.

[33] English BK, Orlicek SL, Mei Z, Meals EA (1997). Bacterial LPS and IFN-gamma trigger the tyrosine phosphorylation of vav in macrophages: evidence for involvement of the hck tyrosine kinase.. J Leukoc Biol.

[34] Manukyan M, Nalbant P, Luxen S, Hahn KM, Knaus UG (2009). RhoA GTPase activation by TLR2 and TLR3 ligands: connecting via Src to NF-kappa B.. J Immunol.

[35] Chen Q, Gupta S, Pernis AB (2009). Regulation of TLR4-mediated signaling by IBP/Def6, a novel activator of Rho GTPases.. J Leukoc Biol.

[36] Shibolet O, Giallourakis C, Rosenberg I, Mueller T, Xavier RJ (2007). AKAP13, a RhoA GTPase-specific guanine exchange factor, is a novel regulator of TLR2 signaling.. J Biol Chem.

[37] Guha M, O'Connell MA, Pawlinski R, Hollis A, McGovern P (2001). Lipopolysaccharide activation of the MEK-ERK1/2 pathway in human monocytic cells mediates tissue factor and tumor necrosis factor alpha expression by inducing Elk-1 phosphorylation and Egr-1 expression.. Blood.

[38] Shi L, Kishore R, McMullen MR, Nagy LE (2002). Lipopolysaccharide stimulation of ERK1/2 increases TNF-alpha production via Egr-1.. Am J Physiol Cell Physiol.

[39] Brook M, Tchen CR, Santalucia T, McIlrath J, Arthur JS (2006). Posttranslational regulation of tristetraprolin subcellular localization and protein stability by p38 mitogen-activated protein kinase and extracellular signal-regulated kinase pathways.. Mol Cell Biol.

[40] Hitti E, Iakovleva T, Brook M, Deppenmeier S, Gruber AD (2006). Mitogen-activated protein kinase-activated protein kinase 2 regulates tumor necrosis factor mRNA stability and translation mainly by altering tristetraprolin expression, stability, and binding to adenine/uridine-rich element.. Mol Cell Biol.

[41] Rutault K, Hazzalin CA, Mahadevan LC (2001). Combinations of ERK and p38 MAPK inhibitors ablate tumor necrosis factor-alpha (TNF-alpha ) mRNA induction. Evidence for selective destabilization of TNF-alpha transcripts.. J Biol Chem.

[42] Herskowitz JH, Siegel AM, Jacoby MA, Speck SH (2008). Systematic mutagenesis of the murine gammaherpesvirus 68 M2 protein identifies domains important for chronic infection.. J Virol.

[43] Pires de Miranda M, Alenquer M, Marques S, Rodrigues L, Lopes F (2008). The Gammaherpesvirus m2 protein manipulates the Fyn/Vav pathway through a multidocking mechanism of assembly.. PLoS One.

[44] Siegel AM, Herskowitz JH, Speck SH (2008). The MHV68 M2 protein drives IL-10 dependent B cell proliferation and differentiation.. PLoS Pathog.

[45] Dunham DM, Arkins S, Edwards CK, Dantzer R, Kelley KW (1990). Role of interferon-gamma in counteracting the suppressive effects of transforming growth factor-beta 2 and glucocorticoids on the production of tumor necrosis factor-alpha.. J Leukoc Biol.

[46] Dean JL, Sully G, Clark AR, Saklatvala J (2004). The involvement of AU-rich element-binding proteins in p38 mitogen-activated protein kinase pathway-mediated mRNA stabilisation.. Cell Signal.

[47] Blackshear PJ, Lai WS, Kennington EA, Brewer G, Wilson GM (2003). Characteristics of the interaction of a synthetic human tristetraprolin tandem zinc finger peptide with AU-rich element-containing RNA substrates.. J Biol Chem.

[48] Sandler H, Stoecklin G (2008). Control of mRNA decay by phosphorylation of tristetraprolin.. Biochem Soc Trans.

