# Supplementary material for: Themis2/ICB1 Is a Signaling Scaffold That Selectively Regulates Macrophage Toll-Like Receptor Signaling and Cytokine Production
Source: PLoS One. 2010 Jul 13;5(7):e11465. doi: 10.1371/journal.pone.0011465 (PMC2903609; doi:10.1371/journal.pone.0011465)
Supplement: Table S1 — Peptides defining Themis2 and Vav. a Mascot ion scores reflect the probability (p) of chance identification of the same peptide: scores>29 for the Themis2 search, or >30 for the Vav search, denote p<0.05. b Scaffold probability reflects the percentage confidence that each peptide sequence identified is a non-random event. The Scaffold calculated probability scores for each protein as a whole was 100%. (0.03 MB DOC) [file pone.0011465.s001.doc]

| ***Themis2 peptides*** | ***MASCOT ion score^a^*** | ***Scaffold™ probability^b^*** |
| --- | --- | --- |
| YFMLSGAYQGK | 42 | 93 |
| KLPASESQAPPPRPPK | 39 | 91 |
| CKPSAPQTLHQILQDPALK | 20 | 68 |
| EFSTAYDLLGALQPGRPLR | 60 | 95 |
| TLEDLVSAMPQNSTRWPIYFK | 38 | 95 |
| VVCEYPETGQTLELNPNFTGLFSPLTSLR | 110 | 95 |
| ***Vav peptides*** |  |  |
| YHLLLQELVK | 65 | 95 |
| ASVNLHSFQVR | 42 | 94 |
| DRSELSLKEGDIIK | 34 | 89 |
