# Supplementary material for: Themis2/ICB1 Is a Signaling Scaffold That Selectively Regulates Macrophage Toll-Like Receptor Signaling and Cytokine Production
Source: PLoS One. 2010 Jul 13;5(7):e11465. doi: 10.1371/journal.pone.0011465 (PMC2903609; doi:10.1371/journal.pone.0011465)
Supplement: Figure S1 — Comparison of murine Themis1 and Themis2. Sequences were compared using the alignment tool in the ExPASy proteomics server (http://us.expasy.org). (0.03 MB DOC) [file pone.0011465.s002.doc]

[Q8BGW0](http://www.uniprot.org/uniprot/Q8BGW0) -MALSLEEFVYSLDLRTLPRVLEIQSGIYFEGSVYEMFGNECCLSTGEVIKITGLKIKKM 59 THMS1_MOUSE

[Q91YX0](http://www.uniprot.org/uniprot/Q91YX0) MEPVPLQDFVSGLDPTSLPRVLRVCSGVYFEGSVYELFGNECCLSTGDLIKVTHVQLQKV 60 THMS2_MOUSE

.:.*::** .** :*****.: **:********:**********::**:* ::::*:

[Q8BGW0](http://www.uniprot.org/uniprot/Q8BGW0) MAEICEGAIGGCESQKPFELPMNFPGLFKVMADKTPYLSIEEITRTVNIGPSRLGHPCFY 119 THMS1_MOUSE

[Q91YX0](http://www.uniprot.org/uniprot/Q91YX0) VCEYPE-------TGQTLELNPNFTGLFSPLTSLRSYRTLEDLVSAMPQNSTRWP-IYFK 112 THMS2_MOUSE

:.* * : :.:** **.***. ::. .* ::*::. :: ..:* *

[Q8BGW0](http://www.uniprot.org/uniprot/Q8BGW0) HLKDIKLENLIIKQGEPIRFNSVEEINGETLVNCGVVRNHQSHSFTLPLSQEGEFYECED 179 THMS1_MOUSE

[Q91YX0](http://www.uniprot.org/uniprot/Q91YX0) STQRIVTKASVVPEDQPLRLEAVEIHHGIRYARCVQVS-KTKELLHLPLSQKGPFWRCKP 171 THMS2_MOUSE

: * : :: :.:*:*:::** :* ..* * : .. : *****:* *:.*:

[Q8BGW0](http://www.uniprot.org/uniprot/Q8BGW0) EHIYTLKEIVEWKIPKNRTRTVKLTDFSNKWDSTNPFPEDFYGTLILKPVYEIQGVLKFQ 239 THMS1_MOUSE

[Q91YX0](http://www.uniprot.org/uniprot/Q91YX0) SAPQTLHQILQDPALKDLTLSCPSLPWN---------------SVILKPQYMLQAIMHMR 216 THMS2_MOUSE

. **::*:: *: * : :. ::**** * :*.:::::

[Q8BGW0](http://www.uniprot.org/uniprot/Q8BGW0) KDIVRILPSLDVEVKDITDSYDANWFLQLLSTDDLFEMTSKEFPVVAEVVEISQGNHLPQ 299 THMS1_MOUSE

[Q91YX0](http://www.uniprot.org/uniprot/Q91YX0) SSIVKIPSTLEVEVEDVTASSQHIHFFKPLRLSEVLAGG-GPFPLTTEILEVPEGPPVFL 275 THMS2_MOUSE

..**:* .:*:***:*:* * : *:: * .::: **:.:*::*:.:* :

[Q8BGW0](http://www.uniprot.org/uniprot/Q8BGW0) S----ILQREKTIVIHKKYQASRILASEIRSNFPKRHFLIPISYKGKFKRRPREFPTAYD 355 THMS1_MOUSE

[Q91YX0](http://www.uniprot.org/uniprot/Q91YX0) SPWVSFLRKGQRLCIYGPASPSWRVVASSKSRKVPRYFMLSGAYQGKLKRRPREFSTAYD 335 THMS2_MOUSE

* :*:: : : *: ..* :.:. :*. *:*::. :*:**:*******.****

[Q8BGW0](http://www.uniprot.org/uniprot/Q8BGW0) LQIAKSRKETLHVVATKAFHT---LHKELSPVSVGDQFLVHHSETTEVVFEGTRKVNVLT 412 THMS1_MOUSE

[Q91YX0](http://www.uniprot.org/uniprot/Q91YX0) LLGALQPGRPLRVVATKDCDGNEEENPDFSFLAVGDRLEVLRSG--QVCGTKGQDIDVLV 393 THMS2_MOUSE

* * . ..*:***** . : ::* ::***:: * :* :* :.::**.

[Q8BGW0](http://www.uniprot.org/uniprot/Q8BGW0) CEKVLN----------------KTREDAQLPLYMEGGFVEVIHDKKQYQISELCTQFCWP 456 THMS1_MOUSE

[Q91YX0](http://www.uniprot.org/uniprot/Q91YX0) CQRLSEQSGEEEEDLEEIEDEAEDKEQILLPLYLSGSFVEEVNDSRRYNLVDLTAQYSLP 453 THMS2_MOUSE

*::: : : :*: ****:.*.*** ::*.::*:: :* :*:. *

[Q8BGW0](http://www.uniprot.org/uniprot/Q8BGW0) FNVKVAVRDLSIKDDILAATPGLQLEEDITDSYLLISDFANPEECWEIPMSRLNMTVRLV 516 THMS1_MOUSE

[Q91YX0](http://www.uniprot.org/uniprot/Q91YX0) CEVKVVTKDTRHPTDPLASFPGLRLEEKLTEPFLVVSLDSQPEMCFEIPPRWLDLTVVEA 513 THMS2_MOUSE

:***..:* * **: ***:***.:*:.:*::* ::** *:*** *::** .

[Q8BGW0](http://www.uniprot.org/uniprot/Q8BGW0) NGSSLPADAGLLQVRSFVEEITEEQYYMMRRYESSLSHPPPRPPKHPSAEEMKLTLLSLA 576 THMS1_MOUSE

[Q91YX0](http://www.uniprot.org/uniprot/Q91YX0) EGQ--PAQVARPLSIAPVEELSEAFYYSLRKLPASESQAPPPRPPKSQGINKKQQNIQSC 571 THMS2_MOUSE

:*. **:.. : ***::* ** :*: :* *:.** * :... : * :. .

[Q8BGW0](http://www.uniprot.org/uniprot/Q8BGW0) EERTINLPKSLKSHHVDRP-------KKLPSDESGQDSRAPVGFQN-------------- 615 THMS1_MOUSE

[Q91YX0](http://www.uniprot.org/uniprot/Q91YX0) KESSVKPQVVEPQKSCPQPQLKAKTLEALPKNKSNVYSKISVHKKDRKPNPQTQNSVLSM 631 THMS2_MOUSE

:* ::: .: :* : **.::*. *: .* ::

[Q8BGW0](http://www.uniprot.org/uniprot/Q8BGW0) ---DVADVERQKSKHGPLQPQAPL-------- 636 THMS1_MOUSE

[Q91YX0](http://www.uniprot.org/uniprot/Q91YX0) KPKTSSSLGKHSTMESHLLPDPDMDDHDYEEI 663 THMS2_MOUSE

:.: ::.: .. * *:. :
