# Supplementary figures and images for: Themis2/ICB1 Is a Signaling Scaffold That Selectively Regulates Macrophage Toll-Like Receptor Signaling and Cytokine Production
Source: PLoS One. 2010 Jul 13;5(7):e11465. doi: 10.1371/journal.pone.0011465 (PMC2903609; doi:10.1371/journal.pone.0011465)

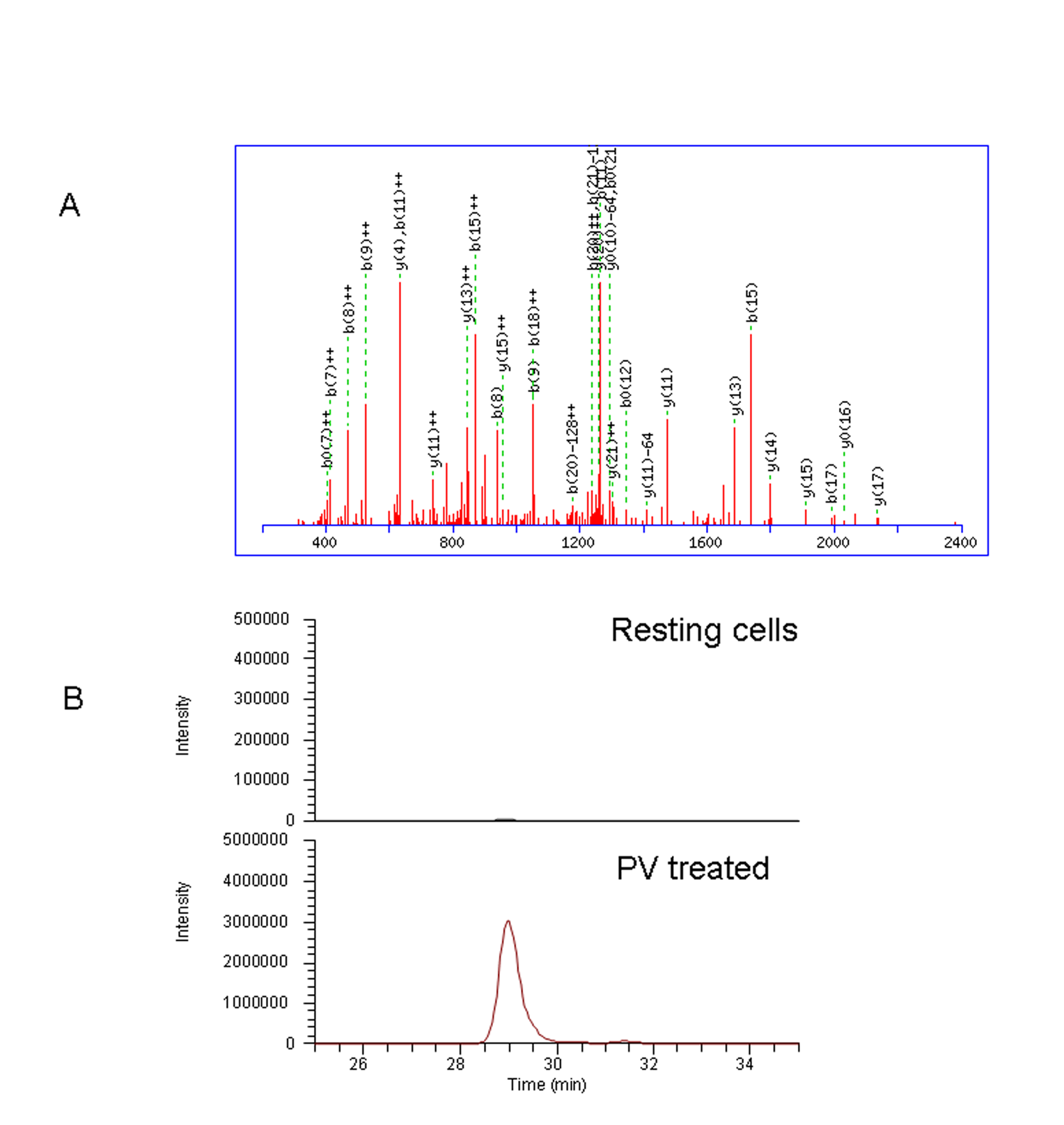

Supplement: Figure S2 — MS/MS detection of tyrosine phosphorylated Y660. Flag-Themis2 from resting cells or pervanadate-treated cells was isolated by immunoprecipiation, separated by SDS-PAGE and digested with trypsin. The tryptic digests were analysed by LC-MS on an LTQ-orbitrap and the c-terminal peptide HSTmESHLLPDPDmDDHDpYEEI was identified from the pervanadate treated sample (A) with a Mascot ion score of 31 (m represents oxidised methionine and pY represents phosphotyrosine). The extracted ion chromatogram of this phosphopeptide ion (m/z = 913.349) from Themis2 isolated from resting and pervanadate-treated cells is shown (B). Similar changes were observed for the same peptide without oxidation (m/z = 902.686) and with one oxidised methionine (m/z = 908.017) ( data not shown). (0.33 MB TIF) [file pone.0011465.s003.tif]

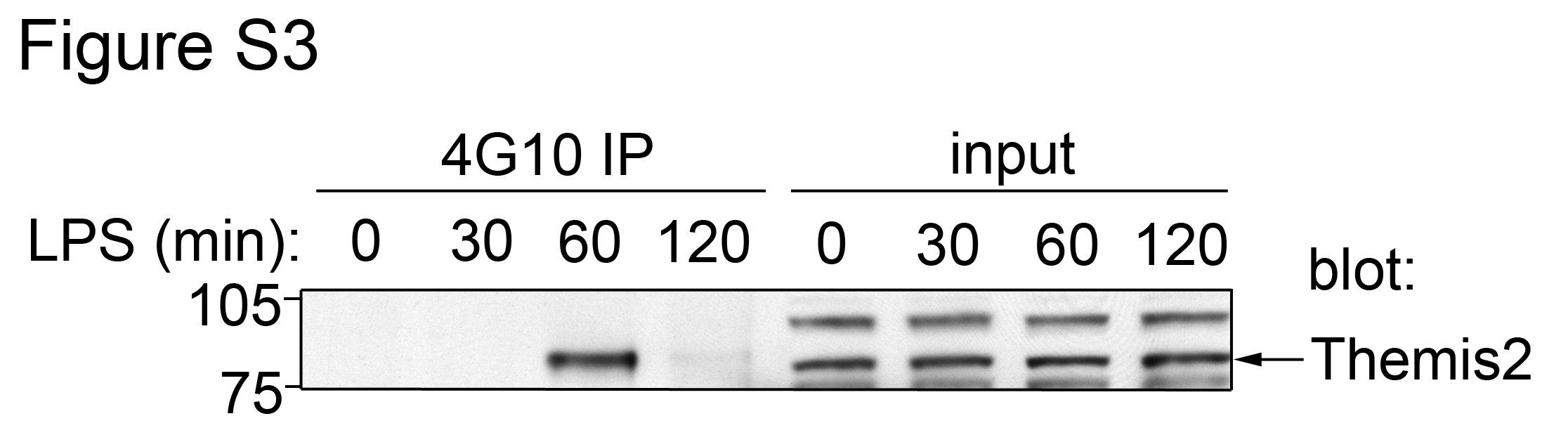

Supplement: Figure S3 — LPS-induced tyrosine phosphorylation of endogenous Themis2. Phosphotyrosine-containing proteins were immunoprecipitated from detergent extracts of LPS-treated RAW cells (10 ng/ml, 0–120 min). Immunoprecipitates or input material from each time point was western blotted for Themis2. (0.16 MB TIF) [file pone.0011465.s004.tif]

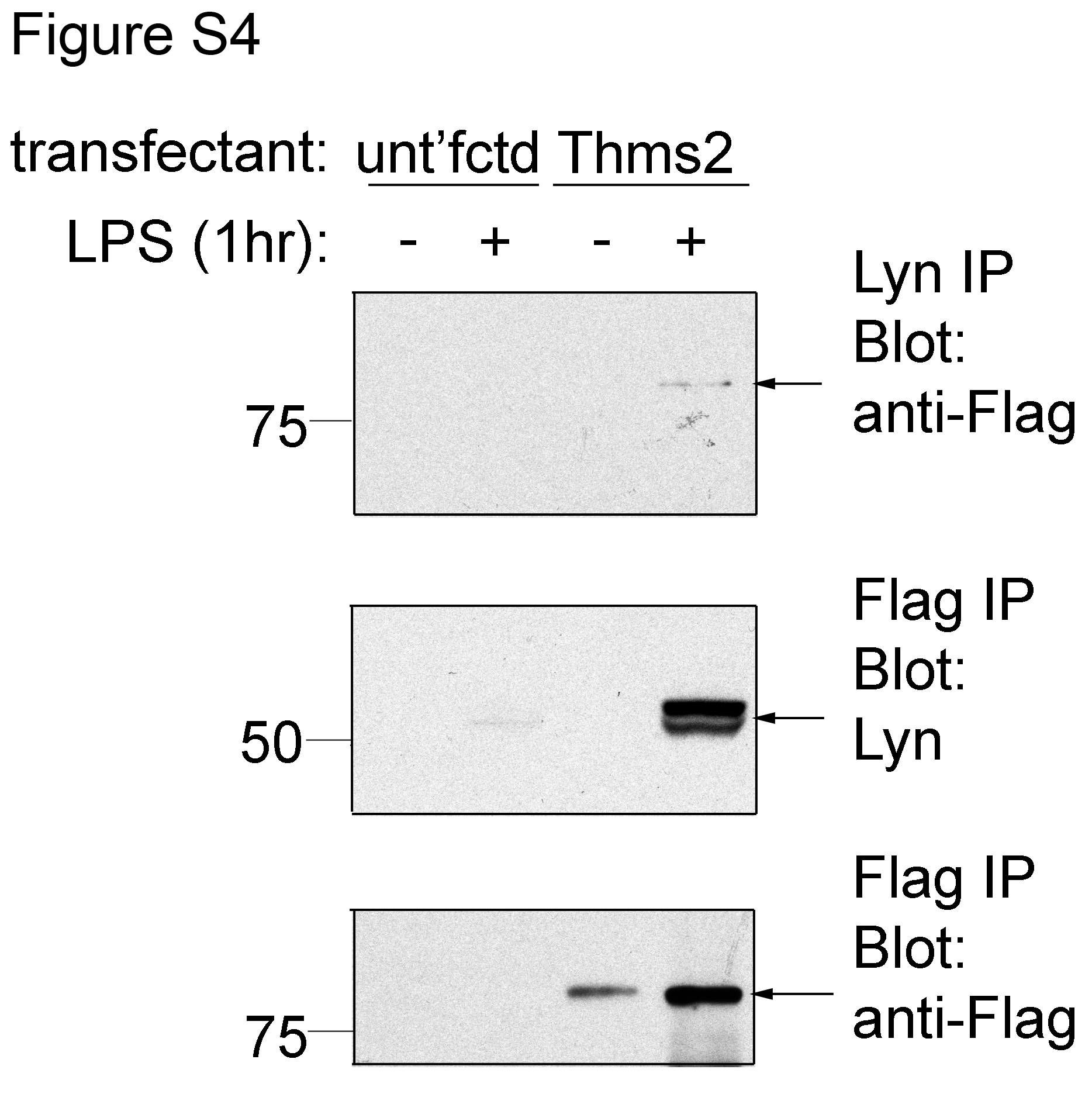

Supplement: Figure S4 — Themis2 interacts with Lyn kinase. Untransfected parental RAW cells or cells stably expressing Flag-tagged Themis2 were stimulated (1 hr), or not, with LPS (10 ng/ml). Immunoprecipitations with anti-Flag or anti-Lyn were performed on separate aliquots of the same cleared extracts. Immunoprecipitates were western blotted as indicated. Data depict a representative of three similar experiments. (0.72 MB TIF) [file pone.0011465.s005.tif]

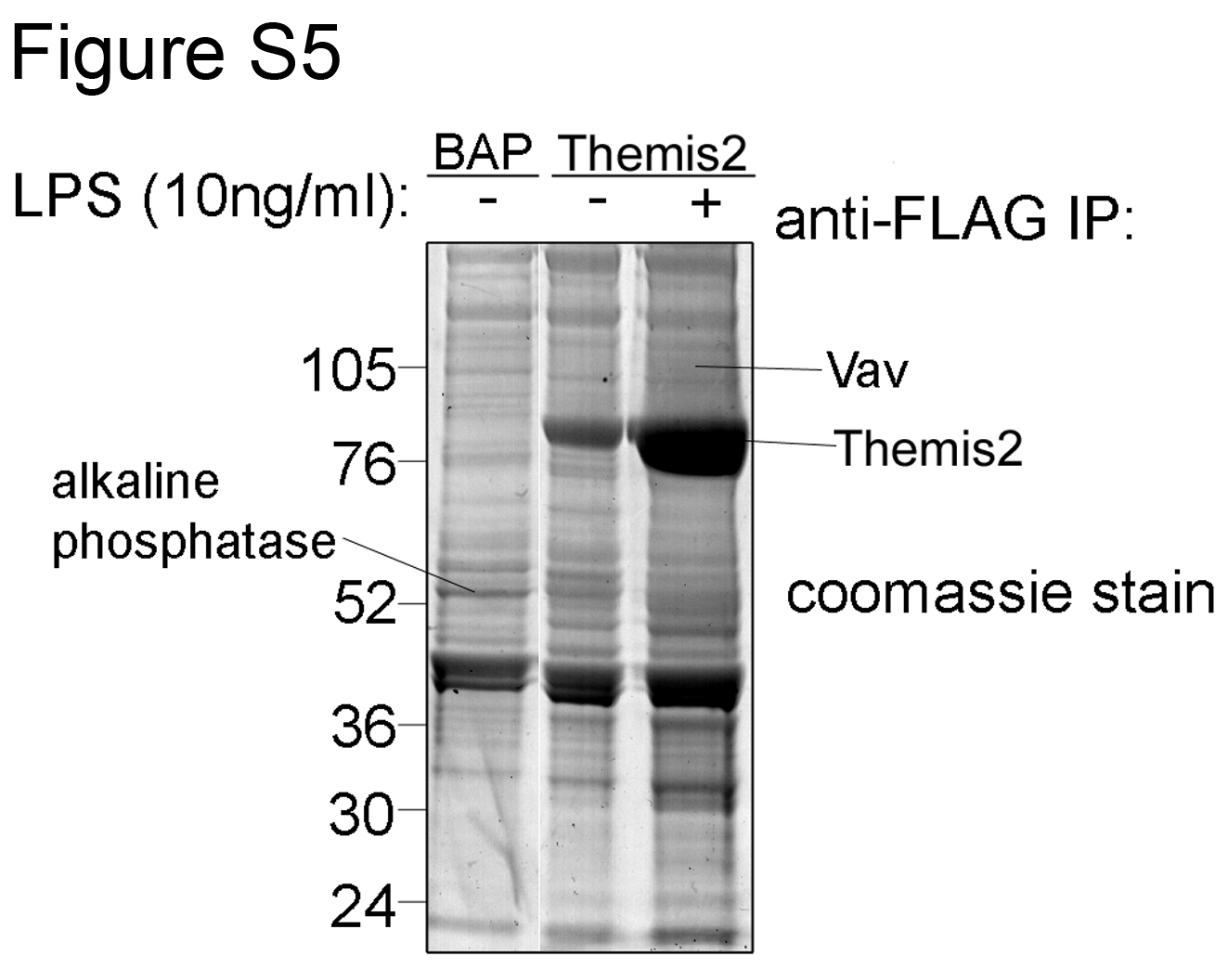

Supplement: Figure S5 — Identification of Themis2 interacting proteins. Flag-tagged and associated proteins were recovered using Flag-agarose beads, eluted, concentrated, resolved and visualised as described in Methods. Protein bands were digested with trypsin and peptides identified by tandem mass spectrometry (see Supplementary Table 1) and validated using Scaffold software. (0.27 MB TIF) [file pone.0011465.s006.tif]

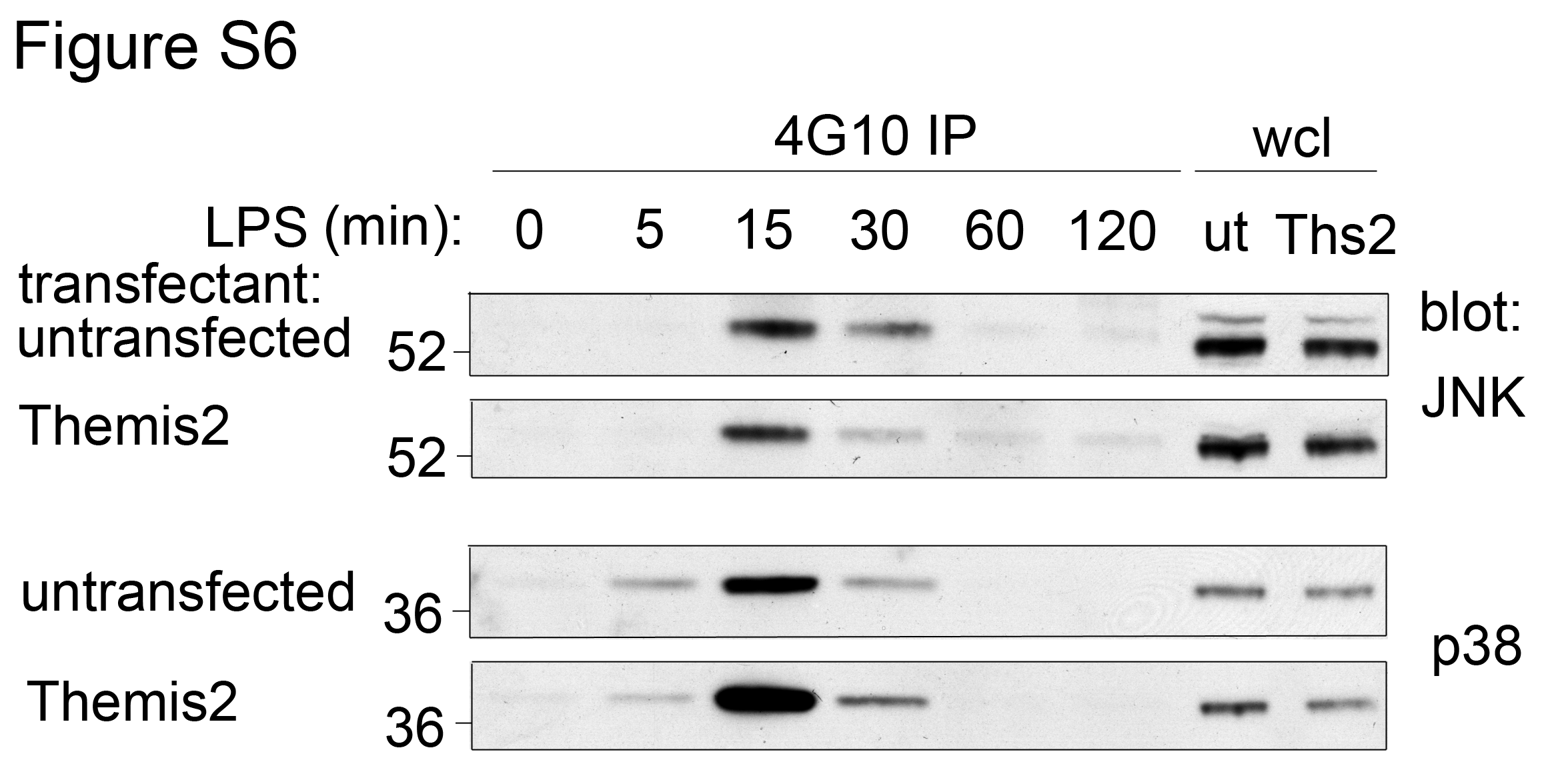

Supplement: Figure S6 — Over-expression of Themis2 promotes LPS-induced p38 but not JNK activation. Parental RAW cells or cells stably over-expressing Themis2 were stimulated with LPS (10 ng/ml) for the period indicated. Anti-phosphotyrosine-containing proteins were immunoprecipitated from detergent lysates with 4G10-agarose beads. The presence of total p38 and JNK MAPKs in immunoprecipitates or input material was detected by western blotting. A representative of five similar experiments is shown. (0.57 MB TIF) [file pone.0011465.s007.tif]

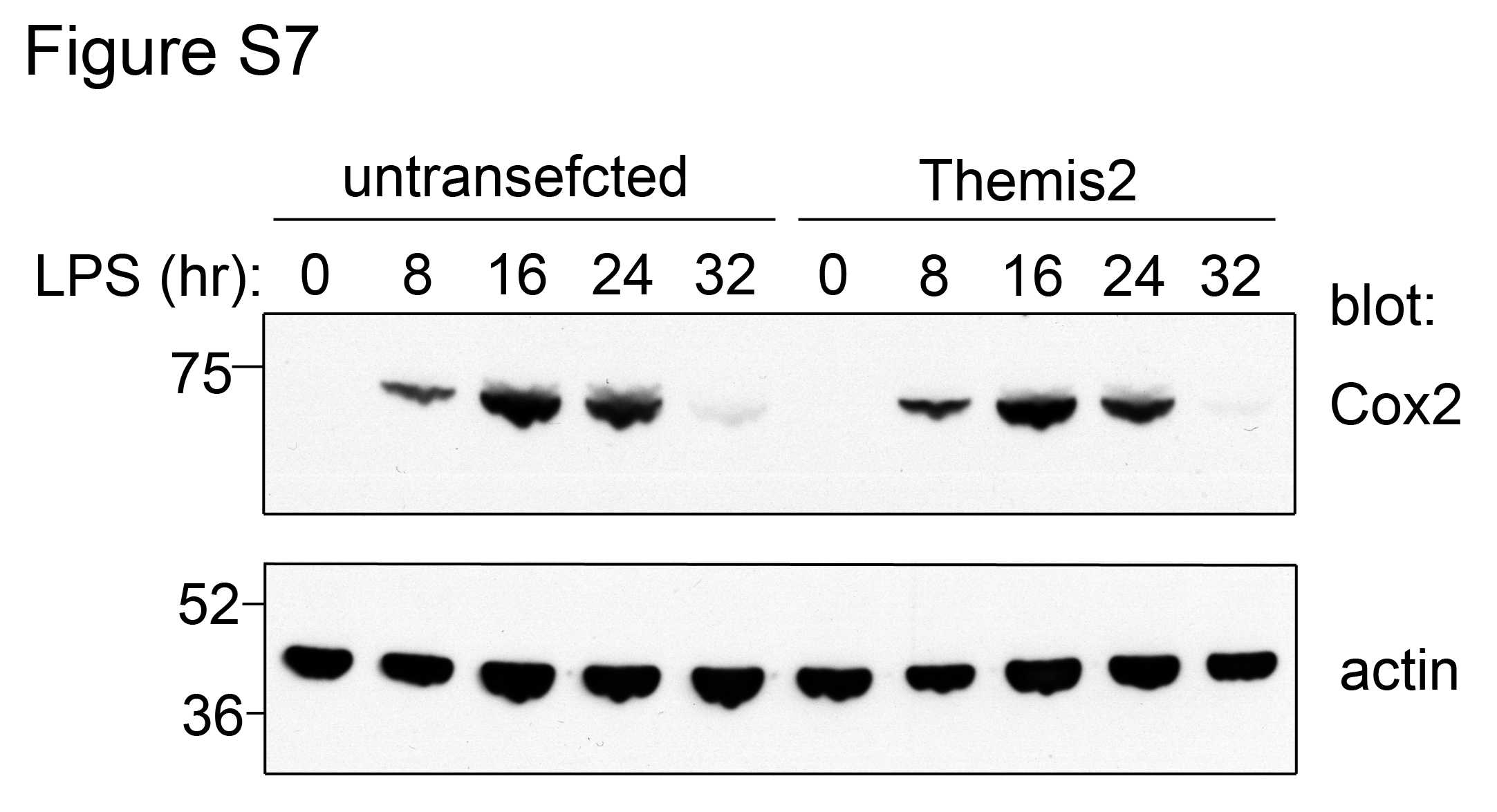

Supplement: Figure S7 — Themis2 over-expression has no effect on LPS-induced Cox2 expression. Matched numbers of parental RAW cells or cells stably over-expressing Themis2 were challenged with LPS (10 ng/ml) for the period indicated and detergent extracts western blotted for Cox2 and actin. Data shown are representative of four similar experiments. (0.61 MB TIF) [file pone.0011465.s008.tif]
